# Gender differences in developmental dyscalculia depend on diagnostic criteria

**DOI:** 10.1016/j.learninstruc.2013.02.004

**Published:** 2013-10

**Authors:** Amy Devine, Fruzsina Soltész, Alison Nobes, Usha Goswami, Dénes Szűcs

**Affiliations:** Department of Psychology, University of Cambridge, UK

**Keywords:** Specific learning difficulties, Mathematics, Prevalence, Sex differences, Developmental dyscalculia

## Abstract

Developmental dyscalculia (DD) is a learning difficulty specific to mathematics learning. The prevalence of DD may be equivalent to that of dyslexia, posing an important challenge for effective educational provision. Nevertheless, there is no agreed definition of DD and there are controversies surrounding cutoff decisions, specificity and gender differences. In the current study, 1004 British primary school children completed mathematics and reading assessments. The prevalence of DD and gender ratio were estimated in this sample using different criteria. When using absolute thresholds, the prevalence of DD was the same for both genders regardless of the cutoff criteria applied, however gender differences emerged when using a mathematics-reading discrepancy definition. Correlations between mathematics performance and the control measures selected to identify a specific learning difficulty affect both prevalence estimates and whether a gender difference is in fact identified. Educational implications are discussed.

## Highlights

► The prevalence of DD ranged from 1 to 6% in a population of 1004 children. ► Reading and maths performance distributions differed for girls and boys. ► No gender difference emerged in the prevalence of DD.

## 1 Introduction

Developmental dyscalculia (DD) is a learning difficulty specific to mathematics claimed to affect between 1.3 and 10% of the population. Different labels have been used in the literature (e.g., mathematics/mathematical/arithmetic learning disability; MLD or ALD, mathematics/arithmetic difficulties; MD or AD). These terms are used interchangeably, but often describe different groups of children. For example, MLD and DD often refer to children with a specific (perhaps biologically-based) disorder of mathematical learning, or number sense (Butterworth, 2005), whereas the terms MD/AD are often used to refer to a larger group of children (the lowest 25–30%) who underperform in mathematics for any of a number of reasons, including environmental factors (Butterworth & Reigosa Crespo, 2007; Mazzocco, 2007). It is important to have clear diagnostic criteria in order to understand the prevalence of DD and also to assess likely genetic origins (e.g., whether x-linked genes may be involved). Here we define DD as a selective impairment of mathematical skills of developmental origin and explore the effects of using different diagnostic criteria on prevalence and gender ratio in a UK population of 1004 children aged 7–10 years.

**Fig. 1:**
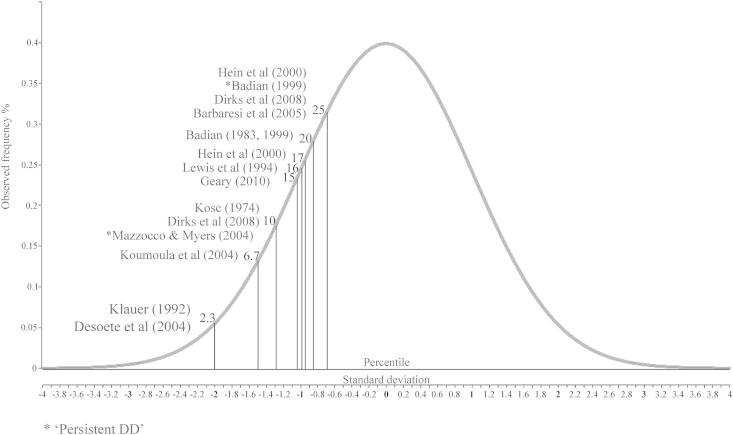
The cutoffs used in DD prevalence studies illustrated on the normal distribution. The percentile scale runs from 0 to 100. Percentile values are shown on top of the normal distribution curve.

As shown in Table 1, the prevalence estimates provided by different demographic studies vary between 1.3% and 10.3% (the mean estimate is about 5–6%). There are some obvious reasons for this broad range of estimates. First, some prevalence studies defined DD using an IQ-achievement discrepancy (e.g., Barahmand, 2008; Barbaresi, Katusic, Colligan, Weaver, & Jacobsen, 2005; Lewis, Hitch, & Walker, 1994; Mazzocco & Myers, 2003), that is, mathematics performance that is substantially below what would be expected given general intelligence. Similarly, Barbaresi et al. (2005) estimated the prevalence of DD using a regression-based discrepancy definition, in which maths performance scores were predicted by a sum of a constant (i.e. a ‘discrepancy’ value) and weighted sum of the IQ score (Barbaresi et al., 2005). Second, others defined DD by the severity of the mathematics impairment using performance cutoffs on standardized tests; the range of cutoffs used in the prevalence studies are represented in Fig. 1. These cutoffs varied broadly, from performance below the 3rd percentile to performance below the 25th percentile (2 *SD* to 0.68 *SD* below the mean). Third, DD has also been defined using a two year achievement delay as a diagnostic criterion, that is, children were categorised as having DD if their mathematics performance was equal to or below the average level of children two years younger (e.g., Gross-Tsur, Manor, & Shalev, 1996; Ramaa & Gowramma, 2002). Fourth, Desoete, Roeyers, & De Clerq (2004) defined DD as children showing resistance to mathematics intervention.

Some demographic studies use control variables in their definitions of DD, such as IQ and/or language abilities. A control variable is necessary to determine whether an abnormality is general to several domains (e.g., it is a general learning disability), or whether it is specific only to mathematics. The use of an IQ-achievement discrepancy definition has been questioned in dyslexia research (Francis et al., 2005) and also represents an important disagreement in DD research. Research has shown that some children with DD may not show an IQ-achievement discrepancy (e.g., Mazzocco & Myers, 2003). Some studies specified average performance in the control measure in their definitions of DD (Desoete et al., 2004; Dirks, Spyer, van Lieshout, & de Sonneville, 2008; Hein, Bzufka, & Neumärker, 2000; Koumoula et al., 2004; Lewis et al., 1994; Ramaa & Gowramma, 2002). Three prevalence studies did measure abilities in other domains but included children with comorbid learning disorders in the DD groups (Gross-Tsur et al., 1996; Mazzocco & Myers, 2003; Ramaa & Gowramma, 2002). Still others report separate prevalence estimates for children with MD only and those with co-occurring reading difficulties (Badian, 1983, 1999; Lewis et al., 1994). Several prevalence studies did not include a control variable at all in their definitions of DD, i.e. they just defined DD/MLD on the basis of low mathematics scores and thus did not differentiate between specific and comorbid learning disabled groups (e.g., Barbaresi et al., 2005; Geary, 2010; Kosc, 1974; Reigosa-Crespo et al., 2011). In the current study we used reading as a control measure. Because we define DD as a learning difficulty specific to mathematics here we did not investigate the comorbidity of mathematics and language impairment; our focus was on mathematics disability not related to language impairment.

We point out that empirical prevalence studies are important because prevalence estimates based on control variables do not simply identify the tail of the normal distribution along a single variable. Rather, because a population is defined on the basis of multiple variables, prevalence values will depend on the strength of the inter-correlation of the specific and the control variable/s (i.e. on the distribution of two or more variables). For example, if DD is defined not only on the basis of mathematics scores but simultaneously on the basis of a control variable like reading achievement, the correlation between mathematics performance and reading achievement must be determined empirically.

The gender ratio of DD reported in past studies has not been consistent; for example, Dirks et al. (2008) reported that the prevalence of DD, defined using performance below the 25th or 10th percentile on Dutch standardized tests, was slightly higher for girls than boys in Grade 4 and 5. In contrast, Barbaresi et al. (2005) found that the cumulative incidence of DD was higher for boys than girls regardless of the age of the children or how DD was defined (see Table 1 for the three definitions they compared). Badian (1983; 1999) found that when DD was defined using performance below the 20th or 25th percentile on the SAT maths the gender ratio was equal for children in lower elementary grades, but the prevalence of DD was higher in boys than girls in Grade 4 and above. Similarly, Mazzocco and Myers (2003) found an equal prevalence of girls and boys with DD in young children (kindergarten to second grade). Other studies reported an equal prevalence of girls and boys with DD in older elementary school children (e.g., Gross-Tsur et al., 1996; Koumoula et al., 2004; Lewis et al., 1994; see Table 1 for definitions used). Ramaa and Gowramma (2002) found that the gender ratio depended on whether DD was diagnosed using a diagnostic test (prevalence higher in boys than girls), teacher identification (prevalence higher in girls) or using exclusionary criteria (equal prevalence). Thus, across the different studies, the gender ratio does not systematically relate to the age of the sample or DD definition used.

Classic studies have shown an advantage for boys in overall mathematics attainment (Benbow & Stanley, 1980). When measuring performance on the mathematics portion of the SAT (SAT-M), Benbow and Stanley (1980) found that boys were overrepresented among the most gifted children (upper 2–5%) of the distribution. Benbow and Stanley (1980) concluded that “male superiority [in mathematics] is probably an expression of a combination of both endogenous and exogenous variables” (p. 1264).

Possible genetic explanations of DD have been suggested by studies showing that specific genetic disorders that are associated with MLD are more frequent in girls than in boys (Fragile X syndrome, Turner syndrome, see Gross-Tsur et al., 1996). The genetic explanation of the gender gap in mathematics can be criticized, for example other developmental factors such as social roles, social expectancies, attribution, motivation, problem-solving strategies and self-confidence may all be non-genetic sources of the gender difference (e.g., Beckwith & Woodruff, 1984; Birenbaum & Nasser, 2006; Kolata, 1980; Nurmi & Aunola, 2005; Spelke, 2005; Tomizuka, Tobias, & Stage, 1981; Timmermans, Van Lieshout, & Verhoeven, 2007). Nevertheless, Benbow, Lubinski, Shea, and Eftekhari-Sanjari (2000) showed that performance in SAT-M predicted professional career 20 years later. Benbow et al. (2000) also reported a follow-up of their 11–13-year-old participants and found that children who were mathematically talented at 11–13 years of age were more likely to have a university degree or a doctorate by 2000. Therefore, the accurate assessment of gender differences in mathematics performance and in DD is critical.

### 1.1 Research questions and hypotheses

We recruited a large sample of British primary school children and investigated the impact of adjusting thresholds and using different criteria on the prevalence and gender ratio of DD. Our research questions were the following: (1) has the prevalence of DD in the UK changed since the last UK prevalence study was conducted 19 years ago (Lewis et al., 1994)? (2) Is the prevalence of DD in the UK comparable to the estimates provided by international studies? (3) What is the gender ratio of DD? (4) Are there gender differences in mathematics and reading performance?

Because previous demographic studies have found different prevalence estimates and have shown mixed results in terms of the reported gender ratio of DD, we did not have specific hypotheses about the prevalence or gender ratio of DD in our British sample (research questions 1–3). With regard to research question (4) we predicted that, in line with Benbow and Stanley's (1980) classic study and the results of recent reports from the Department of Education (Department for Education, 2011a, Department for Education, 2011b), boys may outperform girls in mathematics. Specifically, we predicted that boys may be overrepresented at the upper end of the mathematics performance distribution compared to girls (Hypothesis 1). In line with an abundance of research showing that girls consistently outperform boys in reading (e.g., DfE, 2011a, 2011b; see Logan & Johnston, 2010 for a review), we predicted that girls' reading performance would be greater than boys' reading performance. Specifically, girls would be overrepresented at the upper end of the reading performance distribution compared to boys, who would be overrepresented at the lower end of the reading performance distribution (Hypothesis 2).

## 2 Method

### 2.1 Sample

We tested 1004 children (526 boys and 478 girls) ages 7 years 4 months–10 years 1 month attending Year 3 (*N* = 806 mean age = 8:1) and Year 4 (*N* = 198, mean age = 9:1) of primary school. The participating schools were state primary schools located in Cambridgeshire (12 schools), Hertfordshire (8 schools) and Essex (2 schools), England. The schools comprised a mix of urban schools and outlying rural schools and the catchment populations of the schools were predominantly lower-middle class. The sample included children with statements of special educational needs (SEN) and whose native language was not English (learning English as an additional language; EAL). These children made up less than 1% of the sample. As the general primary population also includes children with SEN and EAL status, we decided to keep these children in the analysis to make our sample more representative of the general population. All children received parental consent to participate. Ethics guidelines prevented us from obtaining the children's school grades or from asking teachers to identify children for the study. The study received ethical permission from the Psychology Research Ethics Committee of the University of Cambridge.

### 2.2 Tests

#### 2.2.1 Mathematics test

The mathematics tests used were the Mathematics Assessment for Learning and Teaching tests (MaLT) (Williams, 2005). The MaLT tests are group-administered written tests. The MaLT tests were developed in accordance with the National Curriculum and National Numeracy Strategy for England and Wales. Test items cover: counting and understanding number, knowing and using number facts, calculating, understanding shape, measurement, and handling data. The MaLT tests were standardized in 2005 with children from 120 schools throughout England and Wales (MaLT 8, *α* = 0.91; MaLT 9, *α* = 0.93). Tests allowed 45 min for completion.

#### 2.2.2 Reading test

We used the Hodder Group Reading Test II (HGRT-II) (Vincent & Crumpler, 2007). The HGRT II level 1 was used for Year 3 pupils, and the HGRT II level 2 was used for Year 4 pupils. These multi-choice tests assess children's reading of words, sentences and passages. The tests were standardized in 2005 with children from 111 schools throughout England and Wales (HGRT II level 1, *α* = 0.96; HGRT II level 2, *α* = 0.95). Each test has two parallel forms which were used in the present study to minimise copying. Tests allowed 30 min for completion.

### 2.3 Procedure

Tests were administered to whole classes between March and December 2010. Classes typically completed both tests in one day, with a break in between the two tests. The order of test administration was counterbalanced across classes.

Children completed the tests under test-like conditions: the children's tables were separated and children were discouraged from speaking or colluding with neighbouring children. At the beginning of the reading test the researchers explained the test instructions and administered two practice questions with the class before the test began. The children worked through the reading test without any input from the researchers or teachers except for explaining the test instructions again where required.

The mathematics assessments do not include practice questions, however the tests allow for invigilators to read the questions if required because the test items require reading and test performance should reflect mathematics ability rather than reading proficiency. Reading questions is also the convention for the administration of National Curriculum mathematics assessments in England and Wales. The test instructions were explained to the children before the test began and invigilators read the questions to the children where necessary.

### 2.4 Data analysis

#### 2.4.1 Analysis of distributions

The relationship of mathematics and reading performance was tested by Pearson's correlations. Gender was controlled for. The normality of distributions of mathematics and reading scores, for all children and for boys and girls separately as well, was tested using the Kolmogorov–Smirnov test. The distributions of mathematics and reading scores were compared to each other by the Mann–Whitney *U* test. If distributions differed, detailed comparisons of the distributions were performed: scores along the distributions were sorted into seven bins, and bin counts were compared by two-sample chi-square tests. The tests were adjusted for unequal sample sizes between boys and girls.

The two-dimensional (mathematics × reading) distribution of scores were also compared between the two genders. In this analysis, 7 × 7 bins (with all the combinations of the bins along mathematics and reading distributions) were compared between boys and girls. The discrepancy between reading and mathematics was also tested, within each gender. Discrepancy scores were calculated by subtracting reading scores from mathematics scores.

#### 2.4.2 Comparison of means

The comparisons were preceded by Kolmogorov–Smirnov tests for each distribution (across gender and domain). Mathematics and reading scores as dependant variables were entered into a repeated measures analysis of variance (ANOVA) with gender (boy or girl) as a between-subject factor and with domain (mathematics and reading) as a within-subject factor. Significant interactions were followed up by Tukey–Cramer post hoc tests. Independent samples *t*-tests were used to compare boys' and girls' scores for the six subtests of the mathematics test (counting/understanding number, number facts knowledge, calculating, understanding shape, measurement, and handling data). To compare the discrepancy scores between genders, the discrepancy scores (mathematics – reading) were entered into an ANOVA with gender as a between-subject factor.

#### 2.4.3 Comparison of correlation values across the distribution

The distribution of reading and mathematics scores was cut into two halves in the following way. Children with mathematics scores from 70 to 104 composed the lower half of the distribution. Children with mathematics scores from 105 to 140 belonged to the upper half of the distribution. The correlation between mathematics and reading scores were computed for both halves, separately. The strength of the correlations between the two halves was compared by the difference test for *r* values. Furthermore, five bins were also created (70–84, 85–98, 99–112, 113–126, and 127–140) from the distribution in order to ensure that any changes in the strength between the two halves of the distribution are due to gradual changes, and not to one or two bins of the distribution having outlier *r* values.

## 3 Results

### 3.1 Distributions of mathematics vs. reading scores and gender differences

**Fig. 2:**
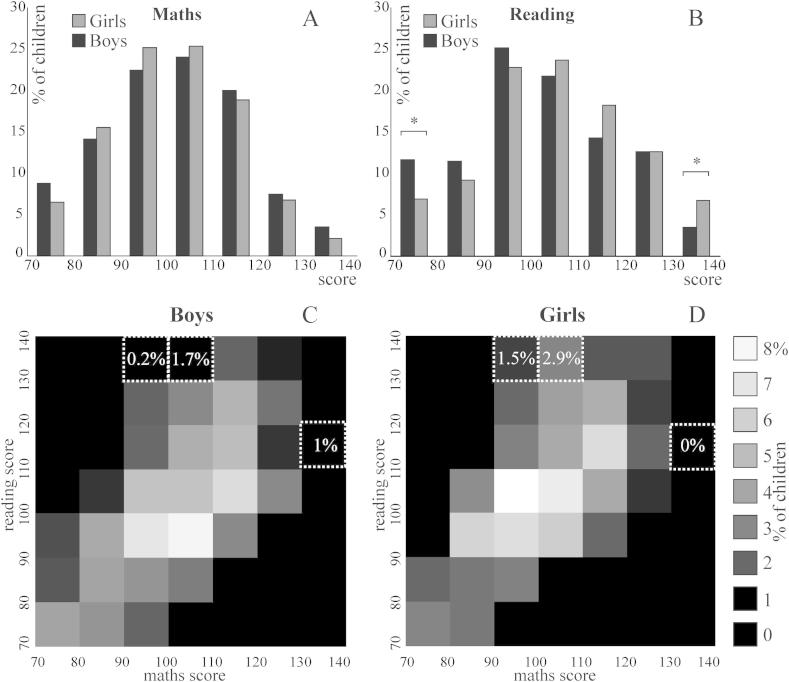
Frequency (%) histograms for mathematics (A) and reading (B). Frequency bins with significant gender differences are marked (see text for exact *p* values). Reading and mathematics score distributions separately for boys (C) and for girls (D). The colour scale represents % of boys and girls (% of children within boys and girls, separately). Frequency bins with significant gender differences are marked (see text for exact *p* values). The percentage of boys and of girls (relative to the number of boys and girls, separately) are indicated within these bins.

Mathematics scores were positively correlated with reading scores (*r* = 0.626, *p* < 0.001) and this correlation remained when controlling for gender (*r* = 0.632, *p* < 0.001). Fig. 2 shows the distribution of mathematics and reading scores across the sample. Neither of the distributions differed from normal (*p* = 0.20 for all).

The distributions of mathematics and reading scores tested separately for boys (*N* = 526) and for girls (*N* = 478) were not different from normal (*p* = 0.21 for both). However, the distribution of reading scores differed significantly between boys and girls (*Z* = −3.31, *p* < 0.001), and the distribution of mathematics scores differed marginally between boys and girls (*Z* = 1.95, *p* = 0.051; the test was adjusted for unequal sample sizes). According to follow-up comparisons, the lower and upper extreme bins in girls and boys differed in reading scores: there were more boys than girls at the lower end and more girls than boys at the upper end of the reading scores distribution, *χ*^2^ (1, *N* = 83) = 5.6, *p* = 0.036 and *χ*^2^ (1, *N* = 64) = 5, *p* = 0.051. None of the bins differed significantly between boys and girls in mathematics.

Fig. 2C and D show the outcome of the two-dimensional distribution comparisons between genders: there was a trend for more girls with average mathematics (90–100, 100–110) and with high reading scores (130–140), *χ*^2^ (1, *N* = 8) = 4.7, *p* = 0.06, *χ*^2^ (1, *N* = 23) = 7.6, *p* = 0.002, and there was a trend for more boys with slightly higher than average (110–120) reading and high mathematics scores (130–140), χ^2^ (1, *N* = 5) = 4.5, *p* = 0.068.

**Fig. 3:**
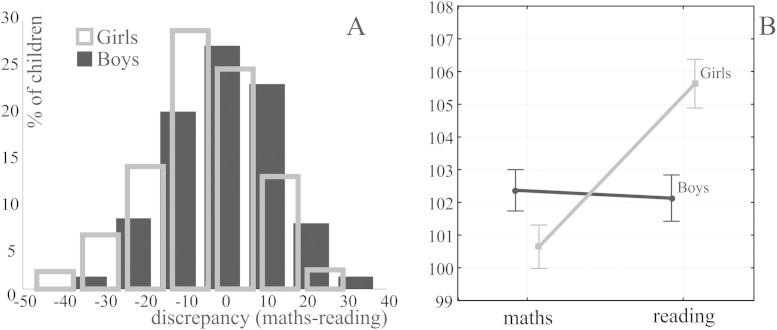
The distribution of mathematics minus reading discrepancy scores (A) and the interaction of gender and domain (B). Bars represent standard error.

The mathematics – reading discrepancy scores were normally distributed ( Fig. 3A, *p* = 0.2 for both). However, the distribution of discrepancy scores differed between boys and girls (*Z* = 5.71, *p* < 0.001). Girls' distribution was shifted to the left, and boys' distribution was shifted to the right, that is more girls had higher reading than mathematics scores while more boys had higher mathematics than reading scores, *χ*^2^ (1, *N* = 533) = 6.81 and *χ*^2^ (1, *N* = 442) = 6.82, *p* = 0.018 for both.

As depicted in Fig. 3B, the domain × gender interaction was significant, *F*(1998) = 25.2, *p* < 0.001. Girls' reading score differed significantly from all other domain × gender cells (*p* = 0.005, *p* = 0.002 and *p* < 0.001 for boys' maths, boys' reading and girls' maths respectively). That is, mathematics and reading abilities differed in girls. In contrast, boys' reading and mathematics scores were not different from each other, *p* = 0.98. Hence, mathematics – reading discrepancy scores differed between girls and boys (girls: −4.98(0.7), boys: 0.29(0.7); *F*(1,998) = 25.2, *p* < 0.001; see Fig. 3A).

**Fig. 4:**
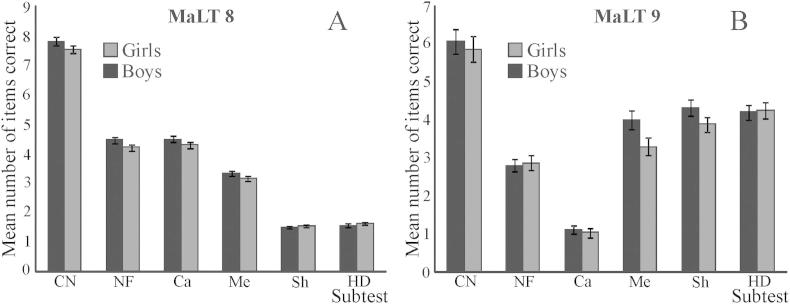
Mean number of items correct on the different mathematics subtests by gender for MaLT 8 (A) and MaLT 9 (B). Bars represent standard error. CN = counting and number, NF = number facts, Ca = calculation, Me = measurement, Sh = understanding shape and HD = handling data.

Scores for the different subtests of the mathematics tests are shown in Fig. 4. Note that subtest scores are presented separately for the MaLT 8 and MaLT 9 tests because these tests contained different numbers of items within each subtest. There were no significant gender differences in performance on any of the subtests.

### 3.2 The effect of criterion levels on the gender ratio of DD

#### 3.2.1 Absolute thresholds

The strength of the correlation of reading and mathematics abilities varied across the distribution. The correlation between reading and mathematics was *r* = 0.57 (*N* = 544) in the lower half of the mathematics distribution (mathematics scores between 70 and 104) and it was *r* = 0.21 (*N* = 440) in the upper half of the mathematics distribution (mathematics scores between 105 and 140). The above correlations differed from each other (difference test: *p* < 0.001). The above change in correlation strength was present in both genders separately as well (boys: *r* = 0.59 (*N* = 304); *r* = 0.16 (*N* = 211), *p* < 0.001; girls: *r* = 0.55 (*N* = 240), and *r* = 0.28 (*N* = 229), *p* < 0.001). The gradual weakening of the correlation was also reflected by decaying *r* values when dividing the distribution into 5 bins in steps of 14 scores: *r* = 0.27, 0.20, 0.18, 0.14, −0.15, from the lowest bin to the highest bin, respectively.

**Fig. 5:**
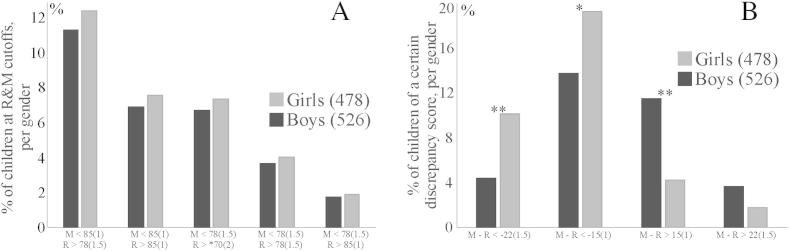
The percentage and number of children in given cutoff cells (A) and the percentage and number of children in given discrepancy score (mathematics – reading) cutoff cells (B). *X*-axis shows the combined mathematics and reading cutoff cells (A) and mathematics – reading discrepancy cutoff cells (B) (the corresponding standard deviation values are in brackets). 5A: * no reading cutoff. 5B: **p < 0.01; and *p < 0.05.

Fig. 5A shows the percentge and number of girls and boys in given cutoff cells. Regardless of the cutoff criteria applied there were no significant differences between the frequency of girls and boys identified as having DD.

#### 3.2.2 Discrepancy (relative) thresholds

Fig. 5B shows the distribution of the sample as a function of discrepancy scores (mathematics minus reading scores). Most children fell within ±1 *SD* difference between mathematics and reading scores (see also Fig. 3A). As can be seen in Fig. 5B, comparisons between genders were significant for all cutoff combinations of the discrepancy scores, *χ*^2^ (1, *N* = 76) = 11.7, *p* = 0.0012, *χ*^2^ (1, *N* = 173) = 4.8, *p* = 0.046, *χ*^2^ (1, *N* = 82) = 24.6, *p* < 0.001, except for a discrepancy of maths-reading of 1.5 *SD* which approached significance, *χ*^2^ (1, *N* = 28) = 4.2, *p* = 0.081. This indicates that there were more girls with better reading than mathematics performance than boys, while there were more boys with better scores in mathematics than in reading, than girls (see also Fig. 3A).

Table 2 shows the frequency of girls and boys defined as having DD using different discrepancies between mathematics performance (maths performance <1 vs. <1.5 *SD* below the mean) and reading performance (reading performance near average vs. >1 *SD* above average). As can be seen in Table 2, the frequency of girls and boys showing particular discrepancies between reading and maths performance was similar. Chi-square analyses confirmed that there were no significant differences between the number of girls and boys identified as having DD using these different discrepancy definitions. Using these discrepancy definitions, the overall prevalence of DD ranged between 0 and 5.3%.

## 4 Discussion

### 4.1 Gender ratio of DD: the impact of mathematics and control variable cutoffs

The empirically measured prevalence of DD depends on both a mathematical criterion variable and on a control variable used to assess the specificity of mathematical weakness. Here *reading* performance served as a control variable. We found that DD prevalence is seriously affected by the cutoff score used to define good *reading* performance. We found that the *prevalence* of DD was the same for girls and boys, regardless of cutoff criteria. Chi-square analyses revealed no significant difference in the frequency of girls and boys for the different cutoff definitions. This finding contrasts with other studies which have reported that the prevalence of DD was slightly higher for girls than boys (e.g., Dirks et al., 2008; Gross-Tsur et al., 1996) or that the prevalence of DD was higher for boys than girls (e.g., Badian, 1983; 1999; Barbaresi et al., 2005; Ramaa & Gowramma, 2002). However, it is in line with other studies that reported an equal prevalence of girls and boys with DD (e.g., Koumoula et al., 2004; Lewis et al., 1994; Mazzocco & Myers, 2003).

On the other hand, if *discrepancy* thresholds are used to define DD (as illustrated in Fig. 5B) gender differences are evident; significantly more girls than boys could be defined as having DD using a discrepancy threshold of 1 *SD* (55 girls vs. 24 boys) or 1.5 *SD* (110 girls vs. 78 boys). However, some of the children who would be defined as having DD using a discrepancy of 1–1.5 *SD* between mathematics and reading performance in fact had mathematics performance which fell within the average range and high reading performance. This profile does not fit a severe impairment of mathematics skills. Rather, these children would be typically regarded as gifted readers rather than weak in mathematics. Therefore we also assessed discrepancy in relative terms, that is, we assessed the number of children who had average or above average reading performance and mathematics performance below 1 *SD* or 1.5 *SD* below the mean (frequencies illustrated in Table 2). Regardless of the relative discrepancy criteria used, there were no significant differences between the frequency of girls and boys defined as having DD. Boys are overrepresented in some other learning disabilities (e.g., reading disability, dyslexia, ADHD and autistic spectrum disorders; Bauermeister et al., 2007; Rutter et al., 2004; Scott, Baron-Cohen, Bolton, & Brayne, 2002), however our data suggest that boys are not under- nor over-represented in DD. The lack of gender difference in DD is problematic for some current genetic theories of DD which suggest a possible role for x-linked genes. However, most of these proposals rely on studies of highly atypical individuals with Fragile X syndrome and Turner syndrome (Kemper et al., 1986; Money, 1973; also see Gross-Tsur, Manor, & Shalev, 1993; Shalev, 2004). In fact, a recent large scale study of mathematical skill in 10-year-old children which using twin data also observed no gender differences (Kovas, Haworth, Petrill, & Plomin, 2007). Hence, we suggest that there is a good chance that gender-related observations from highly special populations are not valid for more typically developing children.

### 4.2 Mathematics/reading performance and gender

The strength of the correlation of reading and mathematics abilities varied across the distribution. The correlation between reading and mathematics was stronger in the lower half of the mathematics distribution than in the upper half of the mathematics distribution and the change in correlation strength was present in both genders.

The distributions of mathematics scores were the same for girls and boys, showing no support for Hypothesis 1. Similarly, mean mathematics scores, and maths subtest scores, did not differ between boys and girls. These findings contrast with Benbow and Stanley's (1980) findings that boys were overrepresented at the higher end of the mathematics performance distribution and recent reports from the Department of Education (DfE) which reported that for children in Key Stage 2 (KS2: Years 3–6 in English primary schools), a higher percentage of boys than girls achieved Level 5 and above in mathematics (corresponding to the upper end of the mathematics performance distribution, Department for Education, 2011a, Department for Education, 2011b). However it is not possible to compare national curriculum statistics directly with the data from the current study, because the children tested here were younger than the age at which KS2 assessments are administered (Year 6).

There are several possibilities for why we did not find a gender difference in mathematics performance here. First, our findings are in line with other research showing that gender differences in mathematics performance are declining, or even non-existent in countries with more gender equal cultures (Else-Quest, Hyde, & Linn, 2010; Guiso, Monte, & Sapienza, 2008). Second, the content of the mathematics test used in our study may have differed from the tests used in other studies. However, the mathematics tests used in past DD studies varied widely due to the fact that the studies were carried out in different countries and in different decades. The maths tests used included standardized assessments (e.g., Stanford Achievement Test-Mathematics, Woodcock-Johnson, Wide Range Achievement Test, Young's Group Mathematics test, Key Math- Revised, Test of Early Math Ability-second edition, Neuropsychological Test Battery for Number Processing and Calculation in Children [NUCALC], and the Cito Rekenen–Wiskunde test) as well as customised test batteries (e.g., those used by Kosc, 1974; Ramaa & Gowramma, 2002). These tests included assessment of numerical operations, conceptual understanding, mathematical reasoning as well as basic number processing. The MaLT mathematics tests includes items assessing all these different areas, therefore the content of the MaLT appears to be similar to the content of tests used in previous studies. Furthermore, the test is also matched to the National Curriculum for England, meaning that the test scores are meaningful in the UK education context.

Third, it is possible that a gender difference in the upper end of the mathematics performance distribution is not evident at Years 3 and 4 (tested here) but emerges at some point between Year 4 and Year 6 (Department for Education, 2011a, Department for Education, 2011b), that is, gender differences in maths performance may emerge towards the end of primary school. As mentioned earlier, several developmental factors have been suggested as non-genetic sources of the gender difference in mathematics performance. Furthermore, negative emotional reactions to mathematics, such as maths anxiety, can develop during the primary school years (Newstead, 1998), and are associated with DD (Rubinsten & Tannock, 2010). Maths anxiety may differentially affect girls' and boys' maths performance (Devine, Fawcett, Szűcs, & Dowker, 2012). Even if girls and boys appear to be performing similarly in mathematics, the development of negative emotional reactions to mathematics warrants attention in the classroom. Overall, educators need to be aware of the ways in which girls and boys differ in terms of the motivational, cognitive and emotional factors associated with learning mathematics. Teachers need to be especially aware of how their own beliefs and emotions regarding mathematics can influence the performance of girls and boys differently (Beilock, Gunderson, Ramirez, & Levine, 2010).

Significant gender differences in the distribution of reading scores emerged: there were more girls in the upper end of the distribution than boys, and more boys in the lower end of the distribution than girls. Moreover, girls' mean reading score was significantly higher than boys' mean reading score. These findings support Hypothesis 2 and are in line with the results of national assessments, which show that girls are overrepresented at the upper end of the reading distribution whereas boys are overrepresented at the lower end of the distribution in Key Stages 1–3 (Key Stage 1: Years 1 and 2 in English primary schools; Key Stage 3: Years 7–9 in English secondary schools; Department for Education, 2011a, Department for Education, 2011b).

There were also gender differences in the distributions of discrepancy scores; that is, the difference between the children's mathematics and reading scores (mathematics minus reading). Girls' discrepancy distribution was shifted into the negative direction, reflecting that girls' performance was better in reading than in mathematics, whereas boys' discrepancy distribution was shifted into the positive direction, reflecting that boys' performance was better in mathematics than reading. These results reinforce the above mentioned gender differences in the reading performance distribution. Although boys' performance was better in mathematics than in reading, the performance advantage in mathematics did not result in boys outperforming girls in mathematics.

### 4.3 Limitations

Our study has some limitations. Due to time and cost constraints related to the large sample size we could not measure the IQ or spelling abilities of the children in our sample. However, similar to the current study, the majority of DD demographic studies used only one control variable (8 studies) and several do not use a control variable at all to determine the prevalence of DD (5 studies). Only one study determined DD prevalence testing two control variables (Lewis et al., 1994). Therefore, we believe that reading ability served as a sufficient control measure in our study according to the procedures adopted by past studies. In addition, of the DD demographic studies with large sample sizes in which IQ was measured, several only administered individual IQ assessments to a subset of their original samples (e.g., Barahmand, 2008; Gross-Tsur et al., 1996; Ramaa & Gowramma, 2002), whereas others accessed IQ information from educational records (Barbaresi et al., 2005) or used a group administered IQ test. For example, Lewis et al. (1994) used the Raven's coloured progressive matrices (Raven, Court, & Raven, 1984) which at the time could be group administered. However, the updated version of Raven's coloured progressive matrices (Raven, 2008) is now individually administered, which was not possible with our large sample. Ethics guidelines prevented us from accessing educational records, so we could not access further information about the children's general abilities or other language skills such as spelling. Further, as the age of our sample was restricted to children between 7 and 10 years of age, our results may not generalise to other age groups. As discussed earlier, UK National Curriculum results have shown gender differences in mathematics performance in slightly older children than the children in our sample. Similarly, the prevalence and gender ratio of DD may differ in children of different ages.

## 5 Conclusions

Prevalence estimates for DD are strongly affected by the inter-correlation between mathematics and the control variable used to determine the specificity of the mathematics impairment. Gender differences in the prevalence of DD did not emerge when using absolute thresholds or relative discrepancy criteria to define DD. Whereas boys are overrepresented in many learning disabilities, the current data suggest that boys are not under- nor over-represented in DD. Hence, our findings suggest that both genders should receive equal attention when assessing dyscalculia, a specific weakness of mathematical ability, in the classroom. We recommend that special attention should be devoted to pupils with average or above average reading performance who show a pronounced weakness of mathematical performance (performance lower than a score of 85 on a standardized mathematics test with an mean of 100). Using these criteria, our study estimated the prevalence of DD as being approximately 6%, consistent with international estimates. Persistent specific weakness can become evident by repeated weak performance on a standardized test (Badian, 1999; Mazzocco & Myers, 2003) and by resistance to intervention (Desoete et al., 2004). At least in our UK sample both genders received equal representation at all performance levels in mathematics but there were more good readers amongst girls than boys. Considered in the context of United Kingdom national data our study raises the possibility that gender differences in maths performance may emerge towards the end of primary school. Were this indeed the case, such a phenomenon would deserve special attention.

## Figures and Tables

**Table 1:** Summary of DD prevalence studies.

First author	Country	Sample	Prevalence	Criteria
Kosc (1974)	Slovakia	375	6.4%	≤10% + control
Badian (1983)	US	1476	3.6%	≤20%
Klauer (1992)	Germany	546	4.4%	<2 *SD*
Lewis et al. (1994)	UK	1056	1.3%	<16% + control
Gross-Tsur et al. (1996)	Israel	3029	6.5%	2 year performance lag + control
Badian (1999)	US	1075	3.9%/2.3%^a^	<20%/<25% a
Hein et al. (2000)	Germany	181/182	6.6%	<17%/<25% + control
Ramaa and Gowramma (2002)	India	251/1408	5.98%/5.54%^b^	Exclusionary criteria/2 year performance lag
Mazzocco & Myers, 2003	US	210	9.6%^a^	≤1 *SD*/<10% + control
Desoete et al. (2004)	Belgium	3978	2.27%/7.7%/6.59%^c^	≤2 *SD* + control + RTI
Koumoula et al. (2004)	Greece	240	6.3%	<1.5 *SD* + control
Barbaresi et al. (2005)	US	5718	5.9%/9.8%/13.8% b	Regression formula; discrepancy formula; <25% + control
Barahmand (2008)	Iran	1171	3.8%	≤2 *SD* + control
Dirks et al. (2008)	Netherlands	799	10.3%/5.6% b	<25%/<10% + control
Geary (2010)	US	238	5.4%	≤15% + control
Reigosa-Crespo et al. (2011)	Cuba	11,652/1966^d^	3.4%	<15%/<2 *SD*^d^

*Note*. Where possible, reported prevalence estimates are for mathematics disability only. RTI = resistance to intervention.

a Persistent DD.

b Prevalence estimates when using the different criteria.

c Prevalence estimates for the Second, Third and Fourth grades respectively.

d Two stage diagnosis.

**Table 2:** Number of children with DD using a certain discrepancy definition, per gender.

	Girls		Boys	
Reading criteria	Maths < 1 *SD*	Maths < 1.5 *SD*	Maths < 1 *SD*	Maths < 1.5 *SD*
Average readers (within 0.5 *SD* mean)	30	4	24	8
High readers (>1 *SD* above mean)	2	0	0	0
